# Telehealth Experience Among Liver and Kidney Transplant Recipients: A Mixed Methods Study

**DOI:** 10.3389/ti.2023.11819

**Published:** 2023-10-16

**Authors:** Dami Ko, Julia Dierker, Rebecca Stouff, Laura Senier

**Affiliations:** ^1^ School of Nursing, Bouvé College of Health Sciences, Northeastern University, Boston, MA, United States; ^2^ Department of Health Sciences, Bouvé College of Health Sciences, Northeastern University, Boston, MA, United States; ^3^ Department of Sociology and Anthropology, College of Social Sciences and Humanities, Northeastern University, Boston, MA, United States

**Keywords:** telehealth, solid organ transplant, healthcare delivery, patient-centered care, mixed methods design

## Abstract

Telehealth has become widely available to solid organ transplant (SOT) recipients during the COVID-19 pandemic. While evidence suggests that telehealth serves as an acceptable alternative for most SOT recipients, their satisfaction and its context remain unclear. This study used a mixed methods approach to investigate the perspectives of SOT recipients (i.e., liver, kidney, and simultaneous liver-kidney) on the benefits and disadvantages of telehealth. A total of 252 adult SOT recipients completed an online survey that quantitatively assessed telehealth experience and satisfaction. Fifteen of them further shared their perspectives by participating in either a focus group or individual interview. Approximately 70% of online survey participants had previously used telehealth for their transplant care. The quantitative data documented that, while recipients were mostly satisfied with telehealth, especially with its effectiveness and convenience, they were less satisfied with the reliability of navigating the telehealth system. The qualitative data further showed that telehealth could be less effective for SOT recipients who perceived themselves as clinically and/or socially vulnerable, needed urgent care, and were concerned about privacy. These findings suggest that the plan for using telehealth to provide transplant care should prioritize personalization, considering unique needs and preferences of each SOT recipient.

## Graphical Abstract

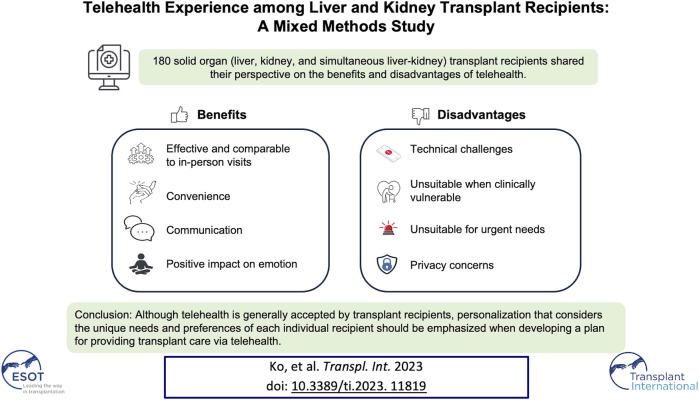

## Introduction

Telehealth refers to the delivery of healthcare, education, and support using telecommunications technologies, such as live videoconferencing [1]. Telehealth has been primarily used to support chronic disease care management that requires regular clinic visits, particularly for individuals living in rural areas [2]. Indeed, telehealth has shown to improve access by reducing travel time and costs [3] while advancing patient outcomes, such as better quality of life and decreased rehospitalization [4]. Despite such benefits, telehealth was not widely available before the COVID-19 pandemic due to multiple barriers, including interstate licensing restrictions, insurance coverage, and lack of infrastructure [5, 6]. The COVID-19 pandemic, however, encouraged many providers and insurers to embrace telehealth.

Solid organ transplant (SOT) recipients, such as liver, kidney, and simultaneous liver-kidney, are required to engage in lifelong care that involves taking immunosuppressants as prescribed and regular follow ups to maintain long-term transplant function. Telehealth has played a critical role in providing essential care for SOT recipients during the COVID-19 pandemic [7]. Telehealth appears to be an acceptable alternative for most SOT recipients. They reported comparable satisfaction to in-person visits with minimized burden of travel [8, 9]. While beneficial in many ways, however, disadvantages of telehealth may exist. Some types of care that require physical contact may not be feasible via telehealth [10]. Lack of technological literacy or reduced access to telecommunications infrastructure among SOT recipients may hinder the effective use of telehealth [9].

Understanding benefits and disadvantages of telehealth from SOT recipients’ perspectives could suggest ways to improve telehealth for them. An approach of continuous improvement is particularly critical because SOT recipients have reported their willingness to use telehealth for certain care services, including synchronous and asynchronous communication with their care providers [11]. While the existing literature has examined the experiences of SOT recipients in relation to transplant care delivered through telecommunications technologies [8, 12–15], evidence is insufficient to fully understand their experiences. Many studies assessed satisfaction of SOT recipients using questionnaires that have not been psychometrically tested [16, 17]. Further, there is a lack of studies employing a mixed method design, which holds the potential to provide a comprehensive understanding of the underlying context influencing their satisfaction. Addressing these gaps in knowledge may inform strategies to advance the telehealth experience among SOT recipients. Thus, this study aimed to understand perspectives of SOT recipients (i.e., liver, kidney, and simultaneous liver-kidney) on the benefits and disadvantages of telehealth.

## Methods

A mixed method design was used to obtain holistic understanding of the perspectives of SOT recipients on telehealth. Quantitative data was collected through an online self-report survey and qualitative data was collected through focus groups and individual interviews.

### Survey Design and Recruitment

Participants were recruited using paid Facebook advertisements between May and August 2021. A series of images and descriptions were used over time to improve the efficacy of the advertising. When a potential participant clicked on the advertisements, they were directed to the study page at Research Electronic Data Capture (REDCap) [18, 19]. Individuals were eligible to participate in the survey if they met the following criteria: (1) were aged 18 years old or greater; (2) had received a liver, kidney, or simultaneous liver-kidney transplant; and (3) were currently receiving care for their transplants at a transplant center located in the United States. Individuals who confirmed that they met all three of these criteria provided online informed consent and completed the survey at REDCap. A total of 876 individuals clicked on the advertisements, 653 were eligible to participate in the study, and 252 individuals completed the survey (response rate 38.6%).

### Measures

All participants were asked if they had used telehealth for transplant care after the United States declared a national emergency due to the COVID-19 pandemic in March 2020. Participants who had not used telehealth were asked to provide reasons for not using telehealth. Those who had used telehealth (*n* = 180) completed a series of questions asking about the use of telehealth. These include types of telehealth, confidence in using telehealth, level of assistance needed to complete telehealth visits, and telehealth satisfaction. Telehealth satisfaction was assessed using the 21-item Telehealth Usability Questionnaire (TUQ), which has strong content validity in assessing the usability of telehealth service [20]. The TUQ has five subscales: usefulness, ease of use, effectiveness, reliability, and satisfaction. Usefulness measures how effective telehealth was at completing desired function. Ease of use determines how easy it was for a patient to complete their appointments and care using telehealth. Effectiveness measures the quality of interaction with clinicians compared to in-person appointments. Reliability measures how well the telehealth system’s online help and feedback was in guiding a patient to navigate the system or correct an error. Satisfaction measures how pleased a patient was with their experience overall. Participants were asked to rate their satisfaction with telehealth they received from their transplant center from 1 (strongly disagree) to 7 (strongly agree). With the TUQ developer’s consent, several items were adjusted to limit the scope of telehealth services provided for transplant care (Supplementary Table S1). Mean total and subscale scores were calculated if at least 75% of the items were answered. Higher scores indicate a greater sense of satisfaction with telehealth care provided. Cronbach’s α coefficients of the scores in this study ranged between 0.75 and 0.97. Demographic and clinical characteristics, such as types of organ transplant and time since transplant, were self-reported by all participants.

### Focus Group and Interview Design and Recruitment

The survey included a question asking if the respondent would be interested in participating in a focus group discussion or interview. A total of 107 survey respondents indicated their willingness to participate and provided an email address. A study team member contacted all 107 respondents to schedule a call. Among them, 92 could not be contacted or withdrew from participation. A total of 15 participants provided online informed consent via REDCap that explained the purpose and procedure of the focus group.

We conducted two focus group discussions between November 2021 and February 2022, each involving four participants. The focus group was moderated by a trained study team member. The focus group moderator’s guide discussed the following topics: quality and connectedness in telehealth compared to in-person visits; participants’ ability to manage their medication and self-monitoring; benefits and challenges of using telehealth; confidentiality; suggestions to improve telehealth; and the pandemic’s influence on utilizing telehealth. The focus group moderator left time for probing, following up, and cross-talk between participants. Focus groups were held via a HIPAA compliant Zoom meeting (Zoom Video Communications, Inc. San Jose, CA) and lasted approximately 60 min. The focus group transcripts were transcribed verbatim and corrected by a study team member who had observed the proceedings.

We also conducted seven individual interviews with survey respondents who could not attend one of the scheduled focus group meetings. Consent and interview procedures matched what was done for the focus groups. Interviews lasted 10–30 min and were recorded and professionally transcribed. The study team member who conducted the interviews corrected the transcripts.

Pseudonyms were used during focus group discussions and individual interviews and in transcripts to protect participant confidentiality. A $30 gift card was given to participants who participated in a focus group or individual interview.

### Data Analysis

We performed quantitative data analysis using IBM SPSS Statistics version 27 (IBM, Armonk, NY). Descriptive statistics were used to describe participant characteristics and scores for the study measures. Sociodemographic characteristics of telehealth users were compared to those of non-users using the Mann-Whitney U test and chi-square test. Associations between characteristics and TUQ scores were assessed using Spearman’s rho correlation and Kruskal-Wallis test. Post-hoc comparisons were conducted using Mann Whitney U test for statistically significant Kruskal-Wallis test results. The level of statistical significance was set at *p* < 0.05. While *p* values were not corrected for multiple tests given the exploratory nature of the study, Bonferroni corrected *p* values were used on *post hoc* pairwise comparisons.

We analyzed the qualitative transcripts following a hybrid inductive-deductive approach [21]. The moderator’s guide and the preliminary codebook we used to analyze transcripts reflected our understanding of the benefits and drawbacks of telehealth for this specific group of patients. We allowed new codes to emerge from the transcripts in response to concepts and themes introduced spontaneously by the focus group respondents, such as perception of risks.

We used NVivo version 12 to code all transcripts. The third author created a codebook that included 29 codes (organized into broad themes), a definition of each code, and representative quotations drawn from the transcripts. The team met to review the codebook and clarify the guidelines for applying codes. The third author then coded one focus group transcript. The team met again to review the coding, to determine if any new codes needed to be added to the codebook, and to review preliminary findings. Once the team reached agreement about codes, themes, and coding guidelines, the third author completed coding all transcripts.

## Results

### Quantitative Results

#### Participant Characteristics

Our full sample of 252 respondents included liver (*n* = 39, 15.7%), kidney (*n* = 198, 79.5%), and simultaneous liver-kidney (*n* = 12, 4.8%) transplant recipients (excluding three missing values, Table 1). Among them, 180 had used telehealth for transplant care. Their median age was 62.0 years (interquartile range [IQR], 55.0–68.0). Most telehealth users were White (87.6%), female (70.8%), and married (57.5%). The majority were either retired (34.1%) or on disability (33.0%) and most had public insurance (44.0%). The median time since receiving their transplant was 55 months (IQR, 22.5–127.5). The characteristics of telehealth non-users were not significantly different than telehealth users, except that telehealth users had higher levels of educational attainment compared to telehealth non-users (Table 1). Among the 72 nonusers, the primary reasons for not using telehealth included: a) telehealth not available at their transplant centers (23.1%), b) not comfortable with technology (16.9%), c) no interest in telehealth (15.4%), and d) no access to telehealth equipment or adequate internet or bandwidth (10.8%).

**TABLE 1:** Participant characteristics.

Characteristics	Frequency (%) or median (IQR)		*p*-value^a^
	Telehealth users^b^ *n* = 180	Telehealth non-users *n* = 72	
Age	*n* = 166	*n* = 61	0.357
	62.0 (55.0, 68.0)	64.0 (55.0, 69.5)	
Race	*n* = 177	*n* = 71	0.137
White	155 (87.6%)	65 (91.5%)	
Black or African American	8 (4.5%)	0 (0.0%)	
Asian	4 (2.3%)	0 (0.0%)	
Other	10 (5.6%)	6 (8.5%)	
Gender	*n* = 178	*n* = 71	0.355
Female	126 (70.8%)	46 (64.8%)	
Male	52 (29.2%)	25 (35.2%)	
Ethnicity	*n* = 175	*n* = 72	0.223
Not Hispanic	169 (96.6%)	67 (93.1%)	
Hispanic	6 (3.4%)	5 (6.9%)	
Education	*n* = 177	*n* = 70	**0.017**
Some high school or high school graduate, a diploma or equivalent (e.g., GED)	18 (10.2%)	26 (22.9%)	
Some college credit, no degree or vocational training	54 (30.5%)	26 (37.1%)	
Associate degree (e.g., AA, AS)	23 (13.0%)	3 (4.3%)	
Bachelor’s degree (e.g., BA, BS)	50 (28.2%)	13 (18.6%)	
Graduate degree	32 (18.1%)	12 (17.1%)	
Marital Status	*n* = 175	*n* = 71	0.098
Single	43 (24.6%)	25 (35.2%)	
Single, living with a partner	7 (4.0%)	4 (5.6%)	
Married	101 (57.5%)	29 (40.8%)	
Widowed	21 (12.0%)	9 (12.7%)	
Separated	3 (1.7%)	4 (5.6%)	
Employment Status	*n* = 176	*n* = 71	0.125
Employed full or part-time	49 (27.8%)	23 (32.4%)	
Retired	60 (34.1%)	27 (38.0%)	
Unemployed	4 (2.3%)	4 (5.6%)	
On Disability	58 (33.0%)	13 (18.3%)	
Other	5 (2.8%)	4 (5.6%)	
Area of Residence	n = 178	n = 71	0.052
City/Urban	47 (26.4%)	28 (38.9%)	
Suburb	68 (37.8%)	17 (23.9%)	
Country/Rural/Small Town	63 (35.0%)	26 (36.6%)	
Miles traveled to visit the transplant center (roundtrip)	*n* = 178	*n* = 72	0.282
0–10 miles	23 (12.9%)	12 (16.7%)	
11–25 miles	34 (19.1%)	18 (25.0%)	
26–50 miles	20 (11.2%)	10 (13.9%)	
51–100 miles	36 (20.2%)	10 (13.9%)	
101–200 miles	35 (19.7%)	7 (9.7%)	
200+ miles	30 (16.9%)	15 (20.8%)	
Income	*n* = 157	*n* = 60	0.174
Less than $20,000	23 (14.6%)	13 (21.7%)	
$20,000 to $34,999	26 (16.6%)	17 (28.3%)	
$35,000 to $49,999	19 (12.1%)	4 (6.7%)	
$50,000 to $74,999	32 (20.4%)	9 (15.0%)	
$75,000 to $99,999	27 (17.2%)	6 (10.0%)	
Over $100,000	30 (19.1%)	11 (18.3%)	
Organ type	*n* = 178	*n* = 71	0.061
Liver	32 (18.0%)	7 (9.9%)	
Kidney	135 (75.8%)	63 (88.7%)	
Simultaneous liver-kidney	11 (6.2%)	1 (1.4%)	
Time since transplant (months)	*n* = 177	*n* = 70	0.555
	55 (22.5, 127.5)	62.5 (30.5, 118.0)	
Insurance coverage	*n* = 175	*n* = 72	0.340
Public	77 (44.0%)	36 (50.7%)	
Non-government insurance/private	42 (24.0%)	14 (19.7%)	
Both	55 (31.4%)	19 (26.8%)	
None	1 (0.6%)	2 (2.8%)	

^a^ Mann-Whitney or Chi-square test.

^b^ Have used telehealth for transplant care.

Note: Bold values denote statistical significance at the *p* < 0.05 level.

Missing values were excluded for calculation of percentages.

#### Telehealth Use

More than half of telehealth users had used multiple types of telehealth (55.3%), with real-time video visits being most common (79.4%; Supplementary Table S2). Over a third of users reported using telehealth for 81%–100% of their visits to their transplant centers over the past 12 months (36.9%). The majority of users rated themselves as very confident in communicating with their provider via telehealth (58.1%) and no assistance needed (87.8%), whereas some users were concerned about the effectiveness of telehealth (34.8%) or reported lack of familiarity or comfortability with the technology (21.2%). Only 13% of telehealth users reported being not likely to use telehealth for transplant care in the future (Supplementary Table S2).

#### Telehealth Satisfaction

**FIGURE 1:**
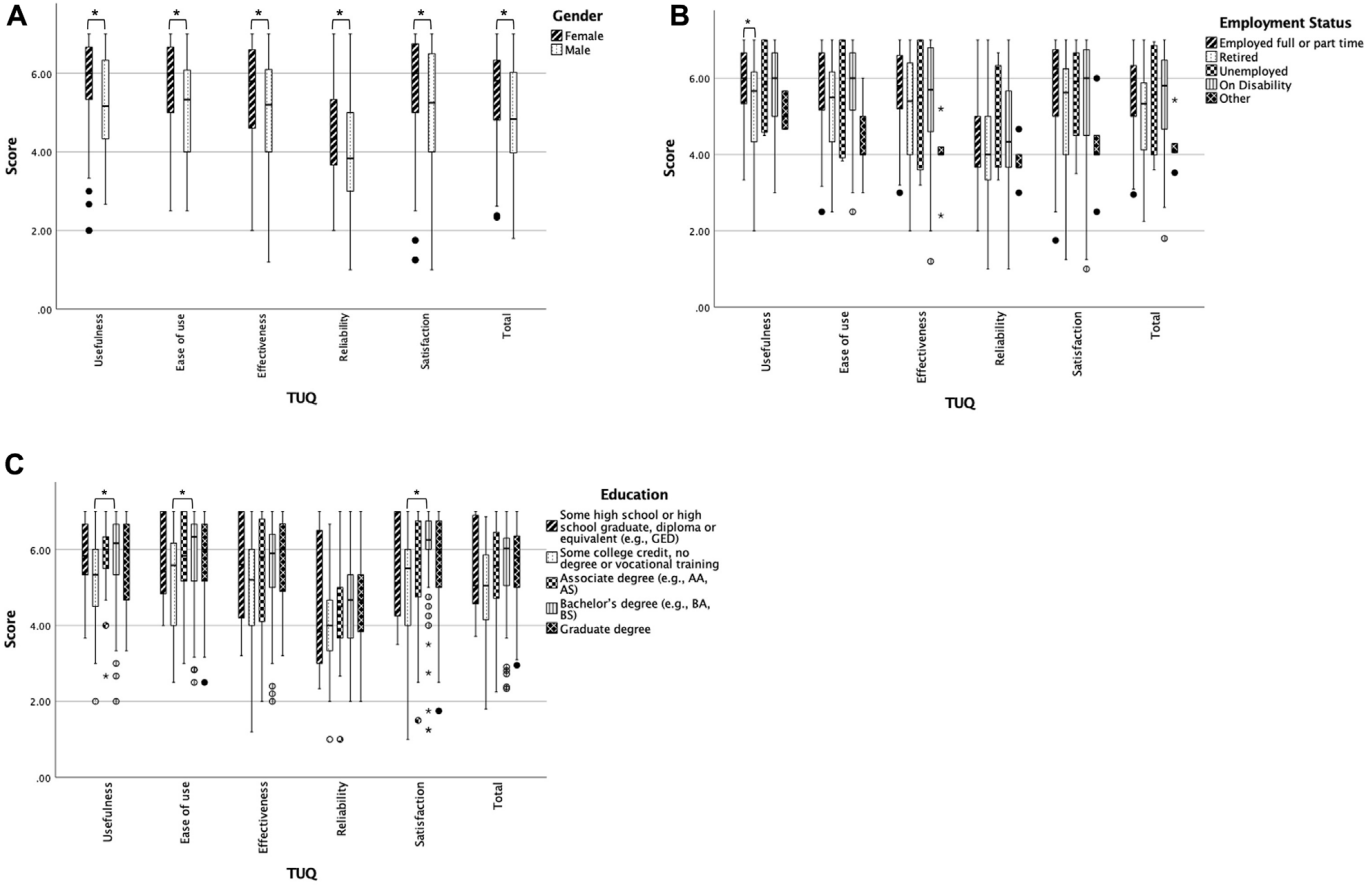
Box plots of telehealth satisfaction by characteristics **(A)** TUQ scores by sex, **(B)** employment status, and **(C)** education. *p* < 0.05 or Bonferroni-corrected *p* < 0.05. The circles are values between 1.5 and 3 IQRs from the end of a box. The stars are extreme outliers indicating values more than 3 IQRs from the end of a box.

The total TUQ scores (Table 2) indicated that transplant recipients were mostly satisfied with telehealth they received for transplant care (Median = 5.6, IQR = 4.5–6.3). The median score of the reliability subscale was the lowest, whereas the median score of the satisfaction subscale was the highest. Table 3 and Figure 1 summarize the statistically significant associations between participants’ characteristics and their telehealth satisfaction. Age and time since receiving transplants were inversely correlated with TUQ total and every subscale scores (*r*_*s*_ = −0.20–−0.29 and *r*_*s*_ = −0.16–−0.21; Table 3). Male recipients had significantly lower median scores in TUQ total and every subscale than female recipients (Figure 1). Recipients who were employed full- or part-time had a significantly higher median score in the TUQ usefulness subscale than those who were retired (Bonferroni-corrected *p* = 0.038; Figure 1). Finally, recipients who obtained some college credit or vocational training had significantly lower median scores in TUQ usefulness, ease of use, and satisfaction subscales than those who had a Bachelor's degree (Bonferroni-corrected *p* = 0.034, 0.049, and 0.017, respectively; Figure 1).

**TABLE 2:** Summary of Telehealth Usability Questionnaire (*n* = 180 telehealth users).

Telehealth usability questionnaire	Median (IQR)
Usefulness, *n* = 180	5.8 (5.0–6.7)
Ease of Use, *n* = 179	5.8 (4.8–6.7)
Effectiveness, *n* = 177	5.6 (4.4–6.6)
Reliability, *n* = 178	4.3 (3.5–5.3)
Satisfaction, *n* = 178	6.0 (4.3–6.8)
Total, *n* = 179	5.6 (4.5–6.3)

**TABLE 3:** Telehealth satisfaction by telehealth user characteristics.

Variables	TUQ					
	Usefulness	Ease of use	Effectiveness	Reliability	Satisfaction	Total
	^a^ *r*_*s*_ (*p-value*)	*r*_*s*_ (*p-value*)	*r*_*s*_ (*p-value*)	*r*_*s*_ (*p-value*)	*r*_*s*_ (*p-value*)	*r*_*s*_ (*p-value*)
Age (*n* = 166)	−0.236 (0.002)	−0.285 (<0.001)	−0.195 (0.012)	−0.230 (0.003)	−0.236 (0.002)	−0.255 (<0.001)
Time since transplant (*n* = 177)	−0.202 (0.007)	−0.173 (0.021)	−0.211 (0.005)	−0.162 (0.032)	−0.213 (0.005)	−0.214 (0.004)

^a^ Spearman ρ correlation coefficients.

### Qualitative Results

#### Benefits of Telehealth

As illustrated in Table 4, focus group participants and interviewees praised the quality and benefits of telehealth. The most frequently mentioned benefit was efficiency, followed by convenience, communication, and affectability. Participants frequently spoke about their satisfaction with telehealth and its benefits not only for managing their transplant recovery but also for other medical care.

**TABLE 4:** Benefits and selected quotes.

Theme–Perceptions of quality		
Sub-themes	Definitions and further subthemes	Quotes
Efficiency	The code was applied to participants’ comments when they spoke about how the appointments were without challenges or problems and utilized time effectively	
		Seamless	“It’s really seamless, because, like, you sign in through the portal, then they route you to, like, a check-in person, and then they route you to the provider.”
Quick		“I, I think they’re faster.”
Convenience	The code was applied to participants’ comments when they spoke about how well telehealth fit with their needs and required little effort from them	
		Location	“But I could do the, I can do telehealth visits when I’m at work, so it does not really matter. I’ve had more than one doctor’s appointment where I’ve been sitting in the field or in my car or something, so…”
Travel		“Pretty good. Um, you know, it, it, it, for in my case it saves me as 45 min one-way drive… And um, again 45 min drive in. Um, you know not too long in the, in the waiting room. The visit was less than 15 min. And uh, you know, I’m driving home again. So a 3 hour day for a, for a 15 min visit. And I, we could have done it over the phone.”
Communication	The code was applied to participants’ comments when they spoke about exchanging and understanding information about their transplant care	
		Comprehensive	“And I would have to say that my visits telehealth are more comprehensive than what I was experiencing, uh, prior to being able to have that.”
Timely		“So, uh, this, this allows a, a doctor and patient or uh, a professional, a medical professional- and patients to communicate very quickly. And with, with very little or, uh, with a great deal of benefit and very little to, um, or very little lost.”
Affectability	The code was applied to participants’ comments as they spoke about how emotionally reactive or negatively affected, they felt before, during or after their transplant appointments	
		Stress	“just to also to add on to what someone said before, not only do you save the stress of not having it drive in and worry about the traffic, not to deal with finding parking. 'Cause some, you know, if I- I’m going into the city…to, to [general hospital] and some, you know, some of those parking lots, it’s like, you feel like you’re in a, um, you’re a car accident waiting to happen as you go. So that’s the benefit of not going in person.”
Mood		“And so, (laughs) this is gonna make me sound like a jerk, but, like, going, like, ha- having to, like, rearrange work schedule, like, stressing out about that and, like, having to go there, it just puts me in a really bad headspace. And I’m, like, annoyed all the time. And, like, I hate sitting in the waiting room and, like, people are there. This whole… It just, it’s, it ruins your day, right?”
Anxiety		“Um, so for me, it’s kind of, it’s nice to have telehealth. But as I said, you know, since I have to have labs every time, it’s not all that helpful to me. But, um, in the in between when he’s wants to give me lab results or whatever, you know, to do it over the phone is a lot easier for me, um, because I have a lot of anxiety when I’m actually, you know, in clinic just because I have such a, I guess a bit of PTSD with things going wrong and such. So it’s easier to, you know, deal with it over the phone and, and ask questions where I’m not so anxious.”

##### Efficiency

Participants repeatedly described their telehealth experience as “seamless.” Many of them noted that it was easier to schedule telehealth appointments with their providers, how smoothly the appointments went, and that the appointments were faster or quicker than an office visit (in part because they and their providers experienced fewer distractions or interruptions during the call).

##### Convenience

Participants frequently listed convenience when we asked them to name the benefits of telehealth. Participants described the convenience of taking their telehealth appointment at any location of their choosing. In addition to talking about joining the appointments at home, participants talked about joining an appointment at work, in their car, or even outside in a field. Participants saw telehealth as particularly beneficial for individuals who might be homebound or are unlikely to seek care. They repeatedly mentioned how they benefitted from telehealth by saving travel time. For example, one respondent reported that a trip to their provider was at least 45 min one way, while another participant was traveling across states for their in-person healthcare, which required days. Another participant, who is blind, said that they found telehealth more convenient because they would not need to arrange travel assistance.

##### Communication

Similar to previous elements, participants spoke positively about the effectiveness and comprehensiveness of their telehealth appointments. Their experiences commonly included mentions of their providers answering all of their questions and communicating in a timely fashion. In most cases, the patients stated that telehealth is especially well suited for visits that are a simple review of lab results or for regular check-in appointments.

##### Affectability

The emotional effects that participants described were mostly positive when talking about the benefits of telehealth. For example, they reported lower levels of stress and anxiety and better moods. Some of this is closely related to the findings described above about convenience; participants said that needing to re-arrange work schedules was troublesome. Finally, some participants said that reviewing lab results could be a stressful part of an appointment, and that being able to reduce some of the stressors (e.g., travel time) allowed them to focus on the content of the conversation.

#### Drawbacks of Telehealth

As illustrated in Table 5, participants identified several drawbacks of telehealth and instances where they perceived it to be riskier than in-person appointments. The most common risk mentioned was perceived clinical vulnerability, followed by urgency, social vulnerability, and privacy.

**TABLE 5:** Drawbacks and selected quotes.

Theme – Perceptions of risks		
Sub-themes	Definitions and further subthemes	Quotes
Perceived Clinical Vulnerability	The code was applied to participants’ comments when they referred to conditions or severity of illness when talking about their perceived risk during telehealth versus in-person appointments	
		Want to be confident during recovery	“uh, but yeah, but for the first 3 months looking back, I mean, at that time I definitely would say, yeah, I wanted to go in because, you know, I did not know yet. I was still, there’s no book, there’s no manual, I mean, they gave me some things to look for- but there is no user manual for a kidney. So, you know, go in and I, I really wanted to, you know, be check and make sure everything was working frequent, you know, working as they expected.”
Concern for clinical errors or important information is missed		“I do not think I would like it, um, at the beginning, because there’s a lot of worry, um, and you kind of need that comforting in-person that everything’s okay, and then, you know, the checking of the labs to prove that everything’s okay. Um, and, you know, the beginning of my transplant was great. But you, I still, you know, I still had constant worry that, you know, things are gonna go wrong. I still feel that way. Um, you know, it’s, it’s kind of a really anxious, kind of, thing to have a transplant. So in those beginning days, I kind of preferred to, you know, actually get to know the doctor in person and communicate in person and have them know me and know my issues and, you know, not just be a person on a screen.”
Changes in health status		“If I go to the portal, I think it, one of the problems with the portal is the fact that, uh, we have not really established a, uh, common denominator for, uh, uh, navigating portals. Uh, for the example, yeah, uh, the other day I was signing up for, doing a pre-visit, uh, online. And uh, there were some medications in there that I, that were not correct. Um, they had me on three mg where I’m on 1.75. But I could not change it. Or even, or even write in about a change or anything. And then there’s no one to talk to say, “Hey, this is. You know, uh, I can’t make a change here that needs really seriously needs to be made because it was one of my immunosuppressant drugs.” So if I was hospitalized or something I do not want them giving me an overdose on, on the immunosuppressants. So um, by, in person I could, I could do that and they can update the chart. Um, where it goes from there, I do not know. But at least I’m more in control of that situation- in person.”
		“Um, so I think it really kind of depends on other health issues. And for me, I would not have wanted, uh, telehealth right after having a transplant.”
		“So I would’ve been fine after probably, like, the second visit to say, you know, “I’ll send my labs in and, and stay home.” But that’s, nothing went wrong, so it might be different. But I think my provider would insist on an in-person, you know, if I were in a situation where they did need to monitor for stuff.”
Urgency	The code was applied to participants’ comments when they described a situation that needed immediate or timely attention	
		Readmitted for urgent care in person	“Most of my problems had, I had to have be readmitted. My lab work was up, um, you know, this test came back abnormal. They would just call me and say, we’re gonna admit you, you’re gonna be a direct admit. Um, so I do not think it would’ve been terribly advantageous for me because I had those problems that needed intervention.”
Social Vulnerability	The code was applied to participants’ comments that spoke about populations that they perceived or they identify with that have challenges with telehealth	
		Age and tech savviness	“I do not think it’s for everybody. I think that certainly, um, like I have a father-in-law that’s 84. He could never use telehealth. He just, even if you were sitting next to him, he, he still would not get the concept of the doctor being there and, and talking to him and that type of thing. So I think it really kind of depends on, um, the patient population and, and how technical savvy they are.”
Impairments		“Um, I’m legally blind, so the, kind of the service that my doctor wanted to go through was not accessible, um, so we e- we end up just doing talking, like, um, you know, just on the phone, because there are some things, um, some programs that are not accessible to, you know, blind individuals or people who use screen readers, things like that.”
Information Processing		“…somebody Zoom meetings me and starts telling me blah, blah, blah. I’m not sure I’m as effective- at listening as when I can see, uh, you know, s- see them face-to-face. So th- there must be some chemical thing going on that you, you know, is in- a little intangible on, on the internet- that, uh, that I’m missing out on.”
Privacy	The code was applied when participants commented that they perceived a risk of privacy or confidentiality when using telehealth	
		Acknowledge some general risk but not too concerned	“They can hack whole hospitals now though (laughs), so- and hold them hostage. So I do not know. They can, they can take the entire Blue Cross’s records and not blink an eye, so I’m not really sure there’s a expectation of privacy anywhere anyway anymore”
“I guess, for me, I’m willing to make the trade-off for the benefits that I get from telehealth.”		
“Wow, I never thought of that 'til you actually just said that		
Has some concern after being asked		Oh my god, maybe I should. But, you know, like, I never thought of that. But you’re right, that’s a good interesting thing. I do not know. Like…'Cause you do hear of stuff like that getting out. And were you ever… Like, have you ever seen a little, like, note on your portal about anything, or has it ever been mentioned…
		It makes me curious. Like, 'cause, you know, you hear of those things happening with other things. Where you least expect it. And I’m like, well… But then again, who the heck’s gonna be trying to look into my… Well, you never know. My health. You know. But, you never know. There’s some crazy people out there, so…But… Yeah. I might have to go look on their site and see what they have to say about that now, yeah. Thanks for bringing that up.”

##### Perceived Clinical Vulnerability

Participants’ comments about the risks associated with the drawbacks of telehealth most frequently touched on their perceived clinical vulnerability as an SOT recipient. In general, participants pointed out that the utilization of telehealth depended on how well they were doing or what issues were present. Their perception of their vulnerability was constantly present due to the second chance that they were given through the transplant. They were aware that their status could change and that their preference for telehealth versus in-person appointments might change in concert with their status. As one participant pointed out, “there is not a manual” for living with an SOT, and some other participants said that in-person appointments helped them to be confident of their recovery progress. Additionally, a few participants shared concerns about the possibility of clinical errors or that something important would be missed.

##### Urgency

Participants noted that telehealth may not be optimal if a problem surfaces in their lab results and they need to be readmitted urgently. If such a visit happened over telehealth, it would still require travel to the hospital or transplant center, thus negating the benefit of telehealth.

##### Social Vulnerability

A participant shared their personal condition of blindness and lamented that their provider’s telehealth videoconferencing platform was not accessible and they had to instead utilize a phone call. Another participant shared a personal need to be in person to adequately process the information from their providers. That experience was not as largely shared by the other participants, but it illuminated a potential subset of people who might need in-person care instead of telehealth to manage transplants. A few participants surmised that elderly patients might be less likely to be “tech savvy,” although technological literacy was not a barrier reported among our participants.

##### Privacy

Perhaps surprisingly, when we asked participants if they had concerns about confidentiality, most said they had not considered this as a potential risk. Even when asked directly about it, they were not concerned about breaches of confidentiality, either on the provider’s side or on their end (i.e., being overhead by a family member or co-worker). One participant acknowledged the potential risk of losing privacy but believed that the benefits of telehealth outweighed it. Finally, one participant, although not concerned before considering the question, became more concerned and stated they needed to find out the privacy protections for their telehealth.

### Connectedness

Finally, there is some evidence in the transcripts that feelings about telehealth varied among participants, depending on the length of time that had passed since their transplant (Table 6). Most interviewees and focus group participants had received their transplant less than 8 years before the pandemic. But there were a couple of participants who had lived with their SOT for decades. Their reports suggest that telehealth might disrupt their relationship with the provider, which they had cultivated over many years. These people mentioned that they had “good” or “easy” rapport with their providers, which in some ways made the shift to telehealth less awkward.

**TABLE 6:** Connectedness and selected quotes.

Theme – Connectedness	
Definition	The code was applied to descriptions of the feeling of connection and trust with their providers or lack of connection
**Sub-themes**	**Quotes**
*Elimination of distractions*	“… I feel like I have more one-on-one attention. There’s not a distraction ‘cause it’s just the two of us in the meeting….I just feel like I get more att- undivided attention, without interruptions.”
*Disruption of established connection during ups and downs of recovery*	“…I have had a lot of setbacks over the last 18 years. Lots and lots. Um, so yeah, I do like seeing them face, you know, face to face too… you know, I, I’ve made friends with these people, you know, over the last 20 years. And it is nice to go in and, and see the group.”
	“I think sometimes just knowing, it’s hard to come, sometimes catch the mood or the personality of the person…And, you know, when a doctor knows you for a length of time, then, you know, a lot of times they say, you know, there’s something that you look different, or-…this just does not seem right according to your labs. By your appearance, or whatever. I think, you know, that’s a big factor.”
*Impediment to establishing trust*	“For me, if I know who you are, I can trust you…Your credentials are very nice, but they do not mean anything to me…So if I can not somehow look at you and size you up in person, um, you’re already at a disadvantage in my head… I go, ‘Okay, I’m not sure who you are, so I’m gonna keep you at the proverbial arm’s length.’
	“…that look on their face, that look in their eye, on the screen, there’s something that’s not there…I do not… Like, uh, you know, I, I do not know how to describe it, but it’s definitely a, a, a less personal experience.”

Despite these generally positive remarks about telehealth, some of these long-term SOT recipients gave several reasons why they nevertheless prefer in-person care. First, they commented that they had received many years of support not only from interdisciplinary transplant team members but from everyone in the transplant center (e.g., receptionists, nursing assistants), and that using telehealth disrupted those connections, which they had established throughout their long recovery. One participant, whose travel to appointments is about 4 hours, said that they viewed these staff members as part of their care team, and that they missed those interactions over telehealth.

Among participants who had had their transplant more recently, some expressed a preference for in-person care, as a means of getting to know their providers and establishing trust. They also spoke about the “chemistry” that can be established when you sit in front of someone and its absence when the appointment is through telehealth. Conversely, the minimization of interruptions and distractions in the telehealth environment could facilitate better connection.

## Discussion

Telehealth has been beneficial for SOT recipients during the pandemic, by minimizing the risk of contracting COVID-19. However, there is a lack of knowledge regarding how satisfied SOT recipients are with their transplant care delivered by telehealth. SOT recipients in this study were mostly satisfied with telehealth, particularly appreciating its efficiency and convenience. Yet, they also expressed concerns that telehealth may not work for everyone.

SOT recipients rated telehealth very highly in its effectiveness to provide comparable quality of care to in-person visits, consistent with previous literature [22, 23]. Particularly, telehealth seems to be a great option for employed recipients compared to retirees due to its convenience and ability to save time and money by reducing the number of trips to their transplant centers. SOT recipients also found telehealth provides a seamless appointment experience while enhancing communication with transplant care providers. They appreciated that they could receive care anywhere that is convenient for them.

This study also, however, suggests that telehealth may not be suitable for all SOT recipients, illustrating the importance of adopting a personalized approach for post-transplant care, based on the patient’s needs and preferences. As demonstrated in previous literature [9, 23], this study identified limited technical capability, lack of online communication skills, and physical disabilities, such as blindness, were possible barriers preventing SOT recipients from the successful use of telehealth. Furthermore, telehealth may not be an ideal care delivery method if recipients perceive their health status as poor. In our study, a small minority of interviewees expressed concern about the limitations of telehealth, including inability to perform physical examination or provide proper care when urgently needed.

Perhaps paradoxically, our study found that patients who had lived with an SOT for decades were most skeptical about the benefits of telehealth, largely because they felt it disrupted a sense of connectedness with transplant care providers, which is crucial to managing a SOT successfully [24, 25]. Established relationships between a patient and healthcare provider facilitates the use of telehealth. Its long-term use, however, may decrease the level of connectedness between patients and providers [9, 26]. Our quantitative and qualitative findings suggest lower satisfaction with telehealth among SOT recipients who undergone SOT a longer time ago; this may indicate that telehealth erodes the sense of connectedness that SOT patients have, not only with their provider, but with all the members of the care team. SOT recipients who have long-lasting, trusted relationships with their transplant care providers may prefer in-person visits over telehealth to maintain such relationships [9, 26]. Further research is needed to identify potential strategies to help establish or maintain the sense of connectedness while providing transplant care via telehealth.

Privacy is one of the common concern individuals may raise when using telehealth [9, 11]. Interestingly, SOT recipients in this study seemed less concerned about privacy. While this finding may indicate their acceptance of telehealth, it may suggest a lack of awareness among recipients about the potential risks of confidentiality breach. For example, our interviewees were relatively unconcerned about the likelihood of a breach on the provider’s side, and none of them seemed concerned that someone in their home or workplace may overhear their conversations. Efforts to broaden use of telehealth should take equity into consideration, since it may not be possible for all recipients to find private space to complete telehealth visits at home or workplace [9, 13]. Moreover, these interviews asked patients about their experience of telehealth during a pandemic and quarantine, and some patients may have considered questions about confidentiality or privacy moot in that circumstance. As healthcare operations return to normal, further research is needed to better understand how SOT recipients perceive the relative risks and benefits of confidentiality and privacy.

A few potential limitations of this study should be noted. While online recruitment offers advantages such as higher participant numbers at a reduced cost compared to traditional methods [27, 28], the small sample recruited online may not represent the broader SOT population. Consequently, the generalizability of the study findings might be limited. Moreover, it should be acknowledged that participants independently assessed their eligibility for participation, but we did not use any validation procedures to evaluate the accuracy of their self-assessment. Finally, the observed correlations do not indicate cause and effect due to the cross-sectional nature of the study.

In conclusion, while SOT recipients readily accepted telehealth during the pandemic, telehealth may not be suitable for all recipients. Clinicians should prioritize assessing a SOT recipient’s needs and preferences when developing a patient-centered transplant care plan. Further research is needed to develop strategies to address potential drawbacks of telehealth.

## Data Availability

The raw data supporting the conclusion of this article will be made available by the authors, without undue reservation.

## Abbreviations

IQR, Interquartile Range; REDCap, Research Electronic Data Capture; SOT, Solid Organ Transplant; TUQ, Telehealth Usability Questionnaire.
