# Supplementary material for: Telehealth Experience Among Liver and Kidney Transplant Recipients: A Mixed Methods Study
Source: Transpl Int. 2023 Oct 16;36:11819. doi: 10.3389/ti.2023.11819 (PMC10613656; doi:10.3389/ti.2023.11819)
Supplement: Supplementary file 1 [file Table1.docx]

**S1. Telehealth Usability Questionnaire**

|  | Strongly disagree | Disagree | Somewhat disagree | Neutral | Somewhat agree | Agree | Strongly agree |
| --- | --- | --- | --- | --- | --- | --- | --- |
| Telehealth improves my access to healthcare services. | 1 | 2 | 3 | 4 | 5 | 6 | 7 |
| Telehealth saves me time traveling to my transplant center. |  |  |  |  |  |  |  |
| Telehealth provides for my healthcare needs. |  |  |  |  |  |  |  |
| It was simple to use the telehealth system at my transplant center. |  |  |  |  |  |  |  |
| It was easy to learn to use the telehealth system. |  |  |  |  |  |  |  |
| I believe I could become productive quickly using the telehealth system. |  |  |  |  |  |  |  |
| The way I interact with the telehealth system is pleasant. |  |  |  |  |  |  |  |
| I like using the telehealth system. |  |  |  |  |  |  |  |
| The telehealth system is simple and easy to understand. |  |  |  |  |  |  |  |
| The telehealth system is able to do everything I would want it to be able to do. |  |  |  |  |  |  |  |
| I could easily talk to the clinician using the telehealth system. |  |  |  |  |  |  |  |
| I could hear the clinician clearly using the telehealth system. |  |  |  |  |  |  |  |
| I felt I was able to express myself effectively. |  |  |  |  |  |  |  |
| Using the telehealth system, I can see the clinician as well as if we met in person. |  |  |  |  |  |  |  |
| I think the visits provided over the telehealth system are the same as in-person visits. |  |  |  |  |  |  |  |
| Whenever I made a mistake using the telehealth system, I could recover easily and quickly. |  |  |  |  |  |  |  |
| The telehealth system gave error messages that clearly told me how to fix problems. |  |  |  |  |  |  |  |
| I feel comfortable communicating with the clinician using the telehealth system. |  |  |  |  |  |  |  |
| Telehealth is an acceptable way to receive healthcare services. |  |  |  |  |  |  |  |
| I would use telehealth services again. |  |  |  |  |  |  |  |
| Overall, I am satisfied with the telehealth system at my transplant center. |  |  |  |  |  |  |  |

Parmanto B, Lewis, Jr. AN, Graham KM, Bertolet MH. Development of the Telehealth Usability Questionnaire (TUQ). Int J Telerehab (2016) 8(1):3–10. doi: 10.5195/ijt.2016.6196.
