# Supplementary material for: Telehealth Experience Among Liver and Kidney Transplant Recipients: A Mixed Methods Study
Source: Transpl Int. 2023 Oct 16;36:11819. doi: 10.3389/ti.2023.11819 (PMC10613656; doi:10.3389/ti.2023.11819)
Supplement: Supplementary file 2 [file Table2.docx]

**S2. Telehealth Use**

| **Types of telehealth used^a^** | **n = 180**  **(cases = 311)** |
| --- | --- |
| Real-time telephone visits | 91 (50.6%) |
| Real-time video visits | 143 (79.4%) |
| Communicate with providers via emails and messages | 75 (41.7%) |
| Other | 2 (1.1%) |
| **Percentage of total visits to a transplant center that was through telehealth over the last 12 months** | **n = 179** |
| 0-20% | 47 (26.3%) |
| 21-40% | 21 (11.7%) |
| 41-60% | 21 (11.7%) |
| 61-80% | 24 (13.3%) |
| 81-100% | 66 (36.9%) |
| **A device used to communicate with providers** | **n = 179** |
| Basic cell phone or Landline | 6 (3.4%) |
| Smartphone | 64 (35.8%) |
| Tablet | 12 (6.7%) |
| Desktop or Portable computer (i.e., laptop) | 23 (12.8%) |
| Other | 0 (0.0%) |
| Multiple | 74 (41.3%) |
| **Confidence in communicating with provider via telehealth** | **n = 179** |
| Not confident at all | 3 (1.7%) |
| Slightly confident | 9 (5.0%) |
| Somewhat confident | 13 (7.3%) |
| Moderately confident | 50 (27.9%) |
| Very confident | 104 (58.1%) |
| **Reasons for less confidence in telehealth^a^** | **n = 66 (cases = 85)** |
| Not very familiar or comfortable with the technology | 14 (21.2%) |
| Inadequate internet or bandwidth issues | 11 (16.7%) |
| lack of knowledge about operating the software or solving problems when a glitch occurs OR lack of support from transplant centers on the use of telehealth system | 11 (16.7%) |
| Bad audio or video quality | 10 (15.2%) |
| Concerned about the effectiveness of telehealth | 23 (34.8%) |
| Concerned about the privacy of telehealth | 11 (16.7%) |
| Do not have access to telehealth equipment | 0 (0.0%) |
| Other | 5 (7.6%) |
| **Level of assistance needed from others to complete telehealth visit** | **n = 180** |
| Cannot complete without assistance | 6 (3.3%) |
| Can complete with little assistance (e.g., partial complete or complete improperly) | 16 (8.9%) |
| Complete with no assistance | 158 (87.8%) |
| **Likelihood of continuing to use telehealth for transplant care** | **n = 178** |
| Not likely at all | 9 (5.1%) |
| Not very likely | 14 (7.9%) |
| Somewhat likely | 47 (26.4%) |
| Very likely | 50 (28.1%) |
| Extremely likely | 58 (32.6%) |

^a^Frequencies of multiple response questions
